# Physiological Noise in Brainstem fMRI

**DOI:** 10.3389/fnhum.2013.00623

**Published:** 2013-10-04

**Authors:** Jonathan C. W. Brooks, Olivia K. Faull, Kyle T. S. Pattinson, Mark Jenkinson

**Affiliations:** ^1^Clinical Research and Imaging Centre, University of Bristol, Bristol, UK; ^2^FMRIB Centre, Nuffield Department of Clinical Neuroscience, University of Oxford, Oxford, UK

**Keywords:** brainstem, physiological noise, fMRI, imaging, 7 T

## Abstract

The brainstem is directly involved in controlling blood pressure, respiration, sleep/wake cycles, pain modulation, motor, and cardiac output. As such it is of significant basic science and clinical interest. However, the brainstem’s location close to major arteries and adjacent pulsatile cerebrospinal fluid filled spaces, means that it is difficult to reliably record functional magnetic resonance imaging (fMRI) data from. These physiological sources of noise generate time varying signals in fMRI data, which if left uncorrected can obscure signals of interest. In this Methods Article we will provide a practical introduction to the techniques used to correct for the presence of physiological noise in time series fMRI data. Techniques based on independent measurement of the cardiac and respiratory cycles, such as retrospective image correction (RETROICOR, Glover et al., 2000), will be described and their application and limitations discussed. The impact of a physiological noise model, implemented in the framework of the general linear model, on resting fMRI data acquired at 3 and 7 T is presented. Data driven approaches based such as independent component analysis (ICA) are described. MR acquisition strategies that attempt to either minimize the influence of physiological fluctuations on recorded fMRI data, or provide additional information to correct for their presence, will be mentioned. General advice on modeling noise sources, and its effect on statistical inference via loss of degrees of freedom, and non-orthogonality of regressors, is given. Lastly, different strategies for assessing the benefit of different approaches to physiological noise modeling are presented.

## Introduction

The human brainstem occupies a key position in the central nervous system (CNS), and is an integral part of the parasympathetic and sympathetic nervous system. The brainstem receives convergent input from spinal and supra-spinal fibers and the cranial nerves, and effectively integrates these signals and coordinates behavioral and physiological responses. As such, this area is of key interest to clinicians and neuroscientists. However, the brainstem is located in an area that suffers from inherently poor signal to noise, due to the close proximity of bone and air-filled cavities, and potent sources of physiological noise. Consequently, functional neuroimaging of this area is problematic. One approach to improving our ability to detect the small blood flow changes associated with neuronal activity, is to account (and correct) for signal changes associated with physiological process that drive signal variation, but may not be related to the neuronal signals of interest.

## Physiological Noise

Physiological noise is generally defined as signal changes in an image that are caused by the subject’s physiology but excludes brain activity of interest (Jezzard, 1999). This excludes scanner-related artifacts, such as ghosting or drift, but can include changes related to rigid-body head motion, although we will not consider these motion changes in the present article. The bulk of the physiological noise signals that we will consider arise from cardiac and respiratory processes.

### Sources of physiological noise

Both the cardiac cycle and the respiratory cycle induce changes in the brain that are detected by MRI. The mechanisms include those due to the cardiac cycle, which induce changes in cerebral blood flow (CBF), cerebral blood volume (CBV), arterial pulsatility, and CSF flow (Greitz et al., 1993; Purdon and Weisskoff, 1998; Dagli et al., 1999; Krüger and Glover, 2001). Mechanisms related to the respiratory cycle include induced changes in the main magnetic field (*B*_0_) (Raj et al., 2001), and changes in arterial CO_2_ partial pressure (Wise et al., 2004). In addition, there are interactions between the cardiac and respiratory systems, such as increased cardiac output during inspiration (the “respiratory pump”; Lin, 1999; Hayen et al., 2012) and changes in CSF flow (Klose et al., 2000).

**Figure 1:**
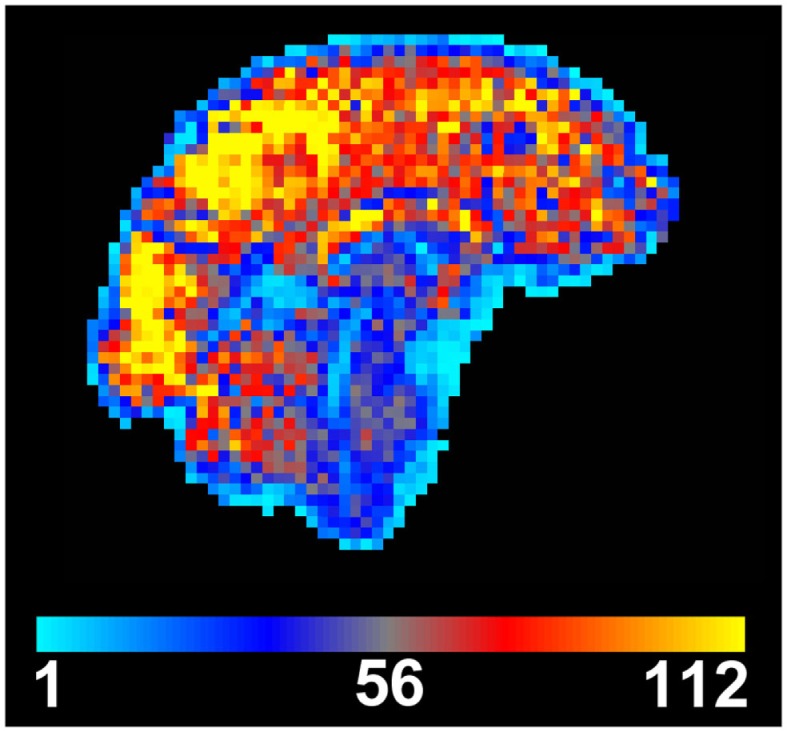
**Temporal signal to noise (tSNR) map created from motion-corrected resting BOLD time series image data**. tSNR is dramatically reduced in the brainstem when compared to other brain areas. Data were acquired from a single subject at 3 T using a 32-channel head coil, with 3 mm isotropic resolution, 100 time points, TE/TR = 30/3000 ms, flip angle = 90**°**, and acceleration factor 2.

The way in which these mechanisms affect the MRI signal also varies. Some changes are due to real movements, such as pulsatility, while some others lead to apparent movement due to varying geometric distortion, associated with changing *B*_0_ fields that are induced by the variable air cavity in the chest. In either case the displacements of voxels in the image cause changes that are particularly strong and noticeable near boundaries between areas with substantially different intensities in the image, such as at the edge of the brainstem (see Figure 1). Other mechanisms create changes in local blood susceptibility, either through oxygenation changes or blood volume changes. These changes will induce signal variations via the BOLD effect, in the same way as those induced by brain activations of interest. There are also mechanisms that affect the signal via changes in tissue composition or via inflow effects.

Many of these noise sources are stronger in the brainstem than in any other part of the brain, due to the increased capacity for movement, due to CSF flow and blood pulsatility, and the closer proximity to the lungs, and hence the stronger induced changes in the *B*_0_ field. Therefore the magnitude and composition of physiological noise in the brainstem is quite different from that encountered in other areas of the brain. The scope of this problem can be easily visualized when looking at a map of the temporal signal to noise ratio (tSNR), which is the ratio of the mean of the time course signal intensity divided by its temporal standard deviation (Parrish et al., 2000). It is clear from Figure 1 that there is an appreciable reduction of tSNR in the brainstem compared to the cerebrum and cerebellum, caused by lower mean signal and/or increased signal variance in the brainstem.

### Characteristics of physiological noise

#### Field strength dependence

Physiological noise increases with the square of the main field strength, whilst signal to noise only increases linearly (Parrish et al., 2000; Krüger and Glover, 2001; Triantafyllou et al., 2005; Hutton et al., 2011). This means that for higher field scanners (e.g., at 7 T) physiological noise can become the dominant source of noise, since it scales in the same way as BOLD contrast. Hence the temporal SNR for functional magnetic resonance imaging (fMRI), and consequently statistical power, does not necessarily improve on higher field scanners, especially in areas where physiological noise is already strong, such as in the brainstem. However, higher field scanners do offer other advantages when scanning the brainstem, such as the ability to have increased spatial resolution, which can be invaluable when investigating small structures.

#### Tradeoffs versus thermal noise and resolution

In MRI, thermal noise is an ever-present source of noise generated by thermal fluctuations within the subject, and to a lesser extent within the receiver electronics, that gives rise to primarily Gaussian-distributed, additive fluctuations in the received signal. When reconstructing images, the distribution of this noise is usually altered, becoming Rician or non-central Chi distributed (Aja-Fernández et al., 2011), although the noise is still reasonably well approximated by a Gaussian noise process in areas of sizeable mean signal magnitude and good tSNR, which are the areas of interest in most fMRI studies. However, whilst the use of array coils and parallel imaging may alter these noise processes, e.g., to a non-central Chi distribution (Breuer et al., 2009), in many cases the Gaussian approximation remains sufficiently good for statistical modeling of the fMRI time series. Further discussion of these issues is beyond the scope of this article.

Although the field strength dependence of thermal-noise-driven SNR is weaker than that of physiological-noise-driven SNR, it is common to scan with higher spatial resolution on high field scanners, which then increases the relative contribution of thermal noise, since thermal SNR scales linearly with the voxel volume. For example, consider 3 mm isotropic fMRI scans acquired at 3 and 7 T. In the 7 T scan the ratio of physiological noise to thermal noise will be 5.4 times higher than in the 3 T scan. However, reducing the voxel volume by a factor of 5.4 times, making it ∼1.7 mm will keep the ratio of physiological noise to thermal noise the same as it was in the 3 mm scan at 3 T. Increasing the resolution further, say to a 1 mm voxel size at 7 T, would then make the ratio (physiological noise to thermal noise) five times smaller in the 7 T scan. Therefore, combined changes in resolution and field strength could mean that the relative ratio of thermal noise and physiological noise was quite different, in either direction, depending on the actual resolutions. As a rough rule of thumb, the contributions of thermal and physiological noise are similar for *cortical* voxels with a 1 mm × 1 mm × 3 mm resolution at 7 T (Triantafyllou et al., 2005). However, in the brainstem, which typically suffers from reduced coil sensitivity due to the distance from (and size of) individual coil elements, there may be an increased contribution of thermal noise at high resolution (as can be seen on Figure 3). This makes it important to check on pilot scans (e.g., by measuring temporal SNR) with different resolutions, even at the same field strength.

#### Effect of imaging sequence

The above comparisons of physiological and thermal noise are all based on using BOLD sensitive fMRI sequences. However, for ASL-based sequences the contributions are likely to be substantially different, since ASL seeks to separate out contributions of blood oxygenation from CBF and CBV. The different sources of physiological noise would therefore contribute quite differently in ASL (see Restom et al., 2006), with oxygenation-related changes being suppressed, blood flow/volume changes being similar or enhanced, and movement-related changes, either real movement due to pulsatility or field-induced image-motion from *B*_0_ changes, having a similar strength. Currently BOLD fMRI has been exclusively employed in studies focused on the brainstem, due to the inherently stronger basic SNR, but the use and characterization of ASL for brainstem imaging deserves further investigation.

## Correction Methods for Physiological Noise

### Acquisition strategies

Several different approaches have been proposed to minimize the influence of physiological noise (including movement) on acquired MR images using acquisition-based strategies. The different techniques broadly fall into three classes: (i) those that attempt to capture images at a fixed point in the physiological process generating noise, e.g., cardiac gating, cardiac-gated multi-echo (ME) acquisitions; (ii) those that attempt to correct images for intensity variation induced by physiological fluctuation, e.g., un-gated ME acquisitions; and (iii) those that acquire additional scans to help identify physiological noise sources from an independent acquisition, i.e., calibration scans. These are discussed below.

#### Cardiac (or respiratory) gating

Cardiac signals appear to be the dominant source of physiological noise in fMRI data obtained from the brain, brainstem, and spinal cord (Dagli et al., 1999; Piché et al., 2009). Previously researchers have attempted to minimize the influence of such signals by recording fMRI data at a fixed point relative to the cardiac cycle, i.e., “gating” (Guimaraes et al., 1998; Malinen et al., 2006). An unavoidable consequence of gating is that the time between consecutive fMRI volume acquisitions is no longer governed by a fixed repetition time (TR) and instead has, e.g., a cardiac cycle dependence, implying that there will be different amounts of longitudinal (T1) relaxation between samples. One approach to this problem is to attempt to correct for the amount of partial saturation of MR signal, via an adjustment based on the time between recorded volumes – the *effective* TR (Guimaraes et al., 1998; Malinen et al., 2006). These methods relying on taking a measurement of the apparent T1 relaxation time for each voxel, and the time between adjacent samples (effective TR) to increase or decrease the measured MR signal appropriately. Whilst this is a potentially attractive technique to use when dealing with structures that suffer from significant cardiac pulsatility, the model relies on perfect registration of adjacent images when determining the apparent T1, and assumes a single relaxation time per voxel, which may be erroneous in the case of partial voluming and imperfect realignment. Another potential confound with these approaches is that as the amount of T1 relaxation between volumes (and therefore measured MR signal) is now dependent on the heart rate (HR), so any stimulus that changes this directly or indirectly (e.g., pain, arousal, fear, hypercapnia, blood pressure) may produce a systematic change in signal intensity that may be independent of the neuronal mechanisms associated with BOLD. The potential for introducing bias in the measurement due to imperfect T1 correction, stimulation-coupled HR changes, and the additional processing required prior to analysis, have led to limited use of this approach.

An alternative approach to correcting for T1 changes induced by cardiac gating is to acquire gated imaging data with multiple echoes per TR (Zhang et al., 2006). By acquiring a minimum of two echoes per TR it is possible to derive an image whose intensity is independent of variable T1 saturation effects, and instead reflects T2^∗^. The image is created by either computing the quotient of the two images (per TR) or by calculating the apparent T2^∗^ using a simple mathematical operation (Beissner et al., 2010). When using this technique, best results have been obtained with careful pre-processing of individual images (e.g., pre-smoothing input data) and optimal normalization to standard templates (Beissner et al., 2011). One potential drawback of this approach is that when modeling evoked BOLD activity obtained using this acquisition method, the time between samples (which is normally fixed) is variable and dependent on the HR, thus an effective (mean) TR is typically used (Beissner et al., 2011). Note that this is primarily a limitation of the statistical modeling packages used for fMRI analysis, which expect data to be sampled at regular intervals. The use of an effective TR and its effect on modeling the BOLD response, and the impact on temporal autocorrelation correction, has yet to be determined. However, it is worth noting that the errors introduced by using an effective TR are expected to be small when used in conjunction with a block design fMRI experiment, but are likely to be more problematic with event related designs.

#### Acquisition-based k- and image-space corrections

By altering sequence parameters, or using modified pulse sequences it is possible to reduce or correct for the presence of physiologically induced image artifacts. It is worth noting that at the relatively long echo times (TE) required for BOLD imaging [$\text{TE}\approx {\text{T2}}_{\text{deoxy(blood)}}^{*}$], imaging data are sensitive to signal dropout and BOLD-like signal changes induced by physiological processes. It is difficult to mitigate the problems related to signal dropout whilst maintaining sensitivity to BOLD. The echo planar (EPI) acquisitions typically used for BOLD imaging are also sensitive to accumulation of phase errors due to variation in static magnetic field (*B*_0_), which is affected by physiological processes (e.g., movement of the lungs). One approach to minimizing these influences was proposed by Pfeuffer et al. (2002), who used a navigator echo to correct for differences in zero-order phase caused by movement of the thorax and abdomen. The changing volume of air in the lungs and position of the tissues surrounding them will induce a time varying (at the breathing rate) change in the main magnetic field (*B*_0_), and the effect of this is to produce a shift in the phase-encoding direction of EPI data (Frank et al., 2001; Raj et al., 2001; Windischberger et al., 2002). By acquiring a navigator echo before the image read-out, and comparing it to the central line of the main k-space data, it is possible to adjust the phase of the acquired data and remove geometric shifts in the phase-encoding direction. It should be noted that this correction will apply uniformly to the imaged slice, and the same effect could be achieved by retrospective motion correction. However, in the case of a segmented EPI acquisition, such a correction will dramatically reduce ghosting in the image.

By combining recent developments in parallel imaging and multi-channel array coils, it is also possible to increase the contrast to noise of EPI data by acquiring ME EPI data (Poser et al., 2006). This approach makes use of the reduced echo train lengths offered with parallel imaging, to (1) reduced susceptibility induced distortions, (2) acquire additional echoes for the same TR, and (3) reduce the apparent through-plane dropout by optimally combining (through weighting each TE image by the contrast to noise ratio) the signal from, in this case, four echoes acquired with an acceleration factor of 2. The advantage of this technique is that non-BOLD contributions to physiological noise, that do not demonstrate strong correlations between the signal measured at each TE, will be reduced via signal averaging. Whilst one might expect physiological processes with typical frequencies of ∼1 Hz (cardiac) and ∼0.3 Hz (respiratory) to demonstrate a strong temporal autocorrelation between the samples in a ME sequence (typically ∼15 ms apart), their contribution appears to not be strongly correlated, perhaps due to local dephasing and blood flow velocity mechanisms, hence echo averaging will be of benefit (Poser and Norris, 2009). Indeed, ME imaging techniques have been used to good effect to minimize physiological signal fluctuation in and around the brainstem (Kundu et al., 2012).

#### Calibration scans

Whilst it may be possible to minimize contributions from physiological noise to acquired fMRI data, through alternate acquisition strategies, it may also be beneficial to identify physiological signals from “calibration scans” and remove them during post-processing of task fMRI scans. Such an approach was proposed by de Zwart et al. (2008), which aims to identify non-task related correlations within a region of interest (e.g., the brainstem) from an additional scan where no task is performed. Correlation between the seed region (e.g., brainstem) and the remainder of the brain is performed, and an arbitrary threshold used to define a mask of these areas (excluding the seed region). During the “real” analysis the time course of signal from the previously determined mask is extracted, orthogonalized (Jezzard et al., 2003) to the experimental design, and included in the GLM to determine activity within the region of interest. Whilst this approach was shown to increase *t*-scores by around 10% in the target brain area, the main disadvantages to such an approach are (1) the need to acquire an additional calibration scan, and (2) the assumption that if the task does produce correlated activity within the predefined mask, then it is safe to consider these signals as noise and remove them from the analysis. Different approaches to achieve the same goal have been proposed, e.g., by using independent component analysis (ICA, see Pre-Processing Strategies) to identify noise sources within fMRI data prior to model estimation using the GLM (Xie et al., 2012).

### Pre-processing strategies

There are a number of strategies for reducing physiological noise that occur after the acquisition but before statistical estimation is performed. These are the strategies that we are classifying as pre-processing strategies.

#### Retrospective correction of k-space data (“RETROKCOR”)

This strategy, originally introduced by Hu et al. (1995), works directly with k-space data, as opposed to the related technique RETROICOR, that works in image-space. RETROKCOR involves correcting the k-space data after acquisition, but prior to reconstruction, by regressing out signals related to the timing and amplitude of the respiratory and cardiac cycles. Therefore it requires the ability to save the k-space data, process this and then apply post-acquisition reconstruction to the processed data.

The principle used here, and in the related method RETROICOR, is to take independent physiological measurements (typically from a pulse-oximeter and respiratory bellows) and create regressors that are used to remove correlated signals from the data. The regressors are based on the timing of the physiological cycles and assuming a periodic but flexible shape. In addition, the respiratory regressors include amplitude modulation, based on the depth of respiration. More information on the creation of regressors can be found in Section “RETROICOR.”

It has been found that RETROKCOR did not perform as well as RETROICOR for standard acquisitions (Glover et al., 2000), but this has not been tested over all types of acquisitions and brain areas. Therefore it is possible that this may be more suitable for methods such as volume-based acquisitions where different k-space lines are associated with distinct times, as opposed to multi-slice images where different slices are associated with different acquisition times. Further investigation of the relative merits of this technique in such circumstances is warranted.

#### Temporal filtering

The cardiac cycle and respiratory cycle have fairly well defined frequencies, being around 1 Hz for cardiac and 0.2 to 0.3 Hz for respiratory processes. Although these are quite variable between subjects they are usually relatively stable within a given subject. Interaction terms also have frequencies defined by the differences of these frequencies, for example, at 0.7 and 1.3 Hz (based on 1.0 ± 0.3 Hz). Therefore it is theoretically possible to remove signals related to these processes by filtering out these frequencies, or a band of candidate frequencies.

In practice, such a temporal filtering approach suffers from a number of problems: (i) the physiological signals also contain harmonics of the base frequencies; (ii) activations of interest may also contain similar frequencies, especially considering the harmonics; and (iii) at typical TR values the sampling rate is too low to uniquely distinguish these frequencies, and they are effectively aliased to different frequencies. The latter problem is the biggest one for typical sequences, as aliasing can only be avoided if the sampling frequency (1/TR) is at least twice as high as that of the highest frequency in the signal, which is the cardiac signal for physiological noise. For example, it requires the TR to be less than 0.4 s in order to avoid aliasing for cardiac signals up to 75 beats per minute.

Aliasing is a fundamental limitation when taking discrete samples and cannot be avoided (see Aliasing or Nyquist Frequency in any standard textbook on signal processing). This limitation effectively means that at least two samples are required within every period to avoid that signal’s frequency being aliased. Otherwise, the signal is undersampled and will have the appearance of a different frequency in the sampled data, which could either be a high frequency, or a low frequency, or something in between. Therefore aliased frequencies are likely to overlap with frequencies from the signals of interest and, consequently, separation by temporal filtering cannot work. However, the emergence of accelerated fMRI acquisitions, such as multi-band fMRI (Feinberg et al., 2010), are making short TR acquisitions feasible without sacrificing brain coverage. With these sequences the use of temporal filtering for short TR acquisitions is becoming a practical option.

Another issue that arises when using temporal filtering, and other pre-processing filtering techniques such as denoising, is correctly accounting for the filtering in the subsequent statistical estimation. Failing to do anything can be problematic if the subsequent statistics are utilizing a form of parametric distribution and making assumptions about the frequency content of the noise. The filtering done here does modify the frequency content of the signal and, in cases where there is substantial filtering (e.g., low pass filtering), this can lead to large parts of the frequency spectrum containing zero power – leading to underestimation of the underlying variance in the data. This is sometimes modeled correctly in parametric statistics, such as is done in certain flexible pre-whitening methods, but would not fit less flexible models such as low-order auto-regressive (AR) models. The implications of this, and also the incorrect statistical Degrees of Freedom (DOF) associated with the estimation, are that the thermal noise influence tends to be underestimated (due to the removal of true signal variance, e.g., with low pass filtering), leading to an increase in false positives. However, it is very difficult to predict the magnitude of the effect on the final results, which may be negligible or substantial, depending on many different aspects of the experimental acquisition, SNR, length, design, etc. To avoid these problems it is often better to employ other, more flexible, statistical methods, such as permutation-based statistics for the higher-level analysis or to perform the equivalent filtering within the statistical estimation, as will be discussed for RETROICOR below (see RETROICOR).

#### Denoising with ICA

Independent Component Analysis is a method of decomposing a dataset into its constituent sources, taking into account the spatial and temporal structure of the sources. In fMRI it is used in a way that enforces spatial independence between component maps but allows time courses in different components to be arbitrarily similar, or different. It has been shown (Beckmann and Smith, 2004) that ICA decompositions are capable of separating out sources of scanner artifact, physiological noise, and brain activation within fMRI. Furthermore, aliasing is not a problem as ICA is still able to distinguish components based on different spatio-temporal patterns in long TR data (Brooks et al., 2008), and thus has an advantage over many of the other strategies, which rely on temporal information alone.

Denoising with ICA is a strategy that relies on identifying unwanted components, such as physiological noise or scanner artifact, and removing them from the dataset prior to further analysis. The difficulty in applying this in practice lies in identifying which components are unwanted or not. The classification of the components can be done manually, or with automated classification tools.

Manual classification of components is subjective and relies on the experience of the person doing the classification. Typically the person inspects the set of components for each subject, which can range from a small number to hundreds, and decides which are noise/artifact that will then be removed. Invariably there are some difficult components where it is hard to tell, and it is known that ICA can produce components that are a mixture of different true sources when the SNR is limited. In such cases it is best to take the conservative approach and leave such components in the data; that is, do not classify them as noise. This ensures that most of the signals related to brain activation remain in the data, minimizing the risk of classifying the signals of interest as noise.

An alternative to this approach, which is particularly useful when considering physiological noise sources, is to compare the spatial location of putative noise components identified from a short TR resting calibration scan (see Calibration Scans), to those estimated from a long TR experimental acquisition (Brooks et al., 2008; Xie et al., 2012). The study by Xie et al. builds on earlier work (Piché et al., 2009) using spatial ICA to identify cardiac components from resting EPI data acquired from the spinal cord, which were classified as noise components on the basis of their location and correlation with recorded physiological traces. Similarly, Xie et al. recorded resting data at short TR (250 ms) to unambiguously identify cardiac and respiratory components, and then compared the obtained spatial maps with ICA results from task fMRI data (acquired with long TR). The components overlapping the noise signals identified on short TR data, could then be included as nuisance regressors in the GLM. This approach was seen to increase both the sensitivity and specificity of spinal fMRI responses to painful electrical stimulation. The main disadvantages to this approach are (1) the need to acquire resting data, and (2) the additional processing steps required to analyze short (resting) and long TR (task) data, and compare the obtained component maps before inclusion in the GLM.

Automated classification methods (Thomas et al., 2002; Tohka et al., 2008; Churchill et al., 2012; Smith et al., 2013a) tend to be based on machine learning approaches that use a training set to learn how to discriminate between different categories of components, such as physiological noise, motion effects, scanner artifacts, and brain activation. As with manual classification, these methods will not be perfect and some errors in classification will occur; for example, the recent method in (Smith et al., 2013a) had an accuracy of over 99% on data from the Human Connectome Project. If the methods provide some control or measure of confidence, it is better to err on the side of not removing certain components when possible. Furthermore, the automated methods rely heavily on the training data matching well with the acquisition being analyzed, and differences in acquisitions (e.g., FOV, field strength, resolution, etc.) can cause the number of mis-classifications to dramatically increase. Therefore, it is important that the classification be carefully monitored, especially when run for the first time on new data. However, these methods do show strong promise in substantially reducing physiological noise.

As mentioned above, care must be taken in the subsequent statistics to account for any denoising done in the pre-processing to avoid falsely inflated statistics. For moderate amounts of denoising this is unlikely to cause a problem for most higher-level analysis methods, where the between-subject variance dominates, but for more extensive denoising this may cause a bias when parametric statistics are used in the higher-level analysis. The precise quantification of these effects is currently unknown and therefore, non-parametric statistics, because they are robust to distributional changes, are currently the preferred option for higher-level analysis of denoised data.

### Physiological noise modeling

As already suggested in the discussion of RETROKCOR (Hu et al., 1995), one method for estimating and removing physiological noise sources from time series fMRI data is to acquire independent physiological measurements from the subject and base the correction on these data. Glover et al. (2000) proposed that this correction was optimally performed in image-space, based on 2D single shot acquisitions using EPI, and was termed RETROspective Image CORrection (RETROICOR).

#### RETROICOR

**Figure 2:**
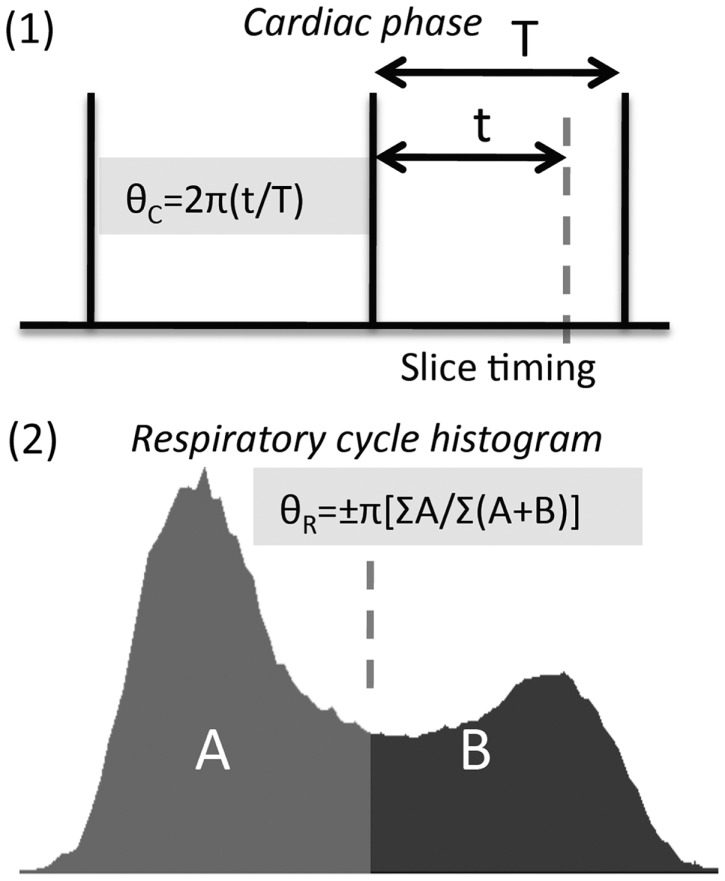
**Calculation of cardiac and respiratory phase for RETROICOR**. (1) Calculation of cardiac phase begins by identifying consistent features from the cardiac trace, e.g., peak in the pulse-oximeter waveform, R-wave in the electrocardiogram (ECG). The timing of slice acquisition relative to these features (vertical lines above) determines the cardiac phase, varying from 0 to 2π. (2) Respiratory phase needs to be calculated differently (see Glover et al., 2000), as both the timing and depth of breathing need to be accounted for, and inspiratory phase distinguished from expiratory phase using a sign change (i.e., phase range is −π to +π).

Typically cardiac and respiratory processes will be monitored using a pulse-oximeter and respiratory bellows, respectively. These physiological waveforms may be recorded on a separate computer, along with scanner triggers to indicate the timing of each volume acquisition. The task is then to determine the phase (cardiac and respiratory) associated with the timing of each acquired slice in the imaged volume. This process is illustrated in Figure 2, see legend for full description.

The phase assigned (varying from zero at the arbitrarily chosen starting point in the cycle, to 2π at the end of the cycle) to each slice, may then be entered into a low-order Fourier expansion (see Glover et al., 2000; Brooks et al., 2008; Harvey et al., 2008), to derive time course regressors that attempt to explain signal changes, which are driven primarily by cardiac or respiratory processes (or their interaction). These signals can then be used to “regress out” physiological signals from the raw time course data as per Glover et al. (2000). Alternatively, the time course of regressors can be included in the GLM – as first suggested by Josephs et al. (1997), whereby the weighting (“beta” or “parameter estimate”) of each component will be adjusted to produce the optimal fit to the data. One advantage of the GLM approach is that it explicitly accounts for the loss of DOF that will occur when including large numbers of nuisance regressors. Equally, if the calculated physiological time courses are not orthogonal to the experimental design (which is likely to be the case), the GLM will apportion the variance between the different regressors, and thus provide a conservative estimate of appropriate statistical quantities, guarding against false positives. This conservative approach is recommended and will automatically happen provided that no user-enforced orthogonalizations of regressors are performed. In relation to the brainstem, Harvey et al. (2008) used hierarchical *F*-tests to determine which regressors explained significant amounts of variance in resting data acquired at 3 T. They concluded that three orders of cardiac, four orders of respiratory, and a single set of interaction terms were sufficient to explain variance in their brainstem fMRI data.

#### Heart rate, respiratory rate, and carbon dioxide

Whilst the cardiac and respiratory phases can explain significant sources of noise in fMRI data, it is also possible to model second order changes associated with variation in the cardiac and respiratory cycles.

Alterations in metabolic rate and ventilation (the product of tidal volume and respiratory rate) have opposing effects upon the partial pressure of carbon dioxide (CO_2_) present in the arterial blood (P_a_CO_2_). CO_2_, due to its vasodilatory effect upon the cerebral vasculature, has a measurable effect on the BOLD signal (Wise et al., 2004, 2007). One approach to correcting for this signal change is to measure the partial pressure of CO_2_ in expired breath (P_ET_CO_2_), and include this measure as an explanatory variable (EV) at the analysis stage (Pattinson et al., 2009a,b). P_ET_CO_2_ varies tightly with P_a_CO_2_ in people with healthy lungs, and is thus considered a valid approximation.

Although it is preferable to directly measure P_ET_CO_2_, in the absence of the necessary monitoring equipment Birn et al. (2006) have proposed a method to approximate changes in P_ET_CO_2_ via a surrogate measure calculated from a respiratory bellows trace: the “respiratory volume per unit time” (RVT, Birn et al., 2006). This method assumes that chest expansion represents measured tidal volume, although this is partly correct, respiratory bellows do not account for lung expansion in the vertical plane (i.e., downwards into the abdomen). Furthermore, changes in metabolism (e.g., induced by drugs) may affect P_a_CO_2_ in a way not fully explained by changes in respiration.

Additionally, variation in HR has also been shown (Shmueli et al., 2007; Chang et al., 2009) to explain “noise” in brain imaging data, however, as with all regressors care should be taken when removing associated signals from fMRI data that may be correlated with the experimental design. For example, in experiments using painful stimuli, HR and the stimulation timing have been shown to be correlated (Tousignant-Laflamme et al., 2005) and in this case including HR in the model has been shown to impact activation statistics (Kong et al., 2009).

#### Alternative model-based approaches

Clearly the RETROICOR approach, and its derivatives, depends on the model chosen to approximate the effect of physiological noise on the measured fMRI signal (although it is still reasonably flexible). To avoid constraining the physiological noise model to a particular set of basis functions (e.g., Fourier basis functions in the case of RETROICOR), it is also possible to use finite impulse response (FIR) functions to model physiological time series data (Deckers et al., 2006). The main assumption with this method is that the physiological processes are quasi-periodic and constant amplitude, and that their effects can be modeled by only using the timing of slice acquisition relative to the peaks detected from the pulse and respiratory waveforms. By finding the peaks in the cardiac and respiratory waveforms, each slice is assigned to a particular “bin” (i.e., a fraction of the cardiac or respiratory cycle). By including separate regressors for each time interval (or bin), whose weight is one (1) for those images falling into the relevant time window, one can build up a complete set of regressors which aims to model the cardiac and respiratory signals. Performance was found to be “at least equivalent to the RETROICOR method” (Deckers et al., 2006). However, there are some issues that arise when using this technique, such as determining the optimal total number of bins (which is somewhat arbitrary and can depend on the fMRI acquisition length, see Kong et al., 2012) also, unlike RETROICOR, FIR-based approaches do not account for the depth of breathing, which has been shown to have a dramatic effect on the induced artifact in EPI data (Raj et al., 2001).

### Improvements in fMRI time series modeling

One way to assess possible improvements of the different correction approaches for physiological noise, is to compare the tSNR calculated from the raw data (motion-corrected only) to that after removing physiological noise.

To illustrate the sort of improvement that might be obtained in the brainstem, we acquired resting fMRI data from two healthy subjects, scanned separately at 3 T (Siemens Verio, manufacturer’s 32-channel head coil) and at 7 T (Siemens Magnetom 7 T, Nova Medical 32-channel Rx with single channel birdcage Tx). Data were acquired using a rectilinear EPI sequence with the following voxel sizes 3 T (3 mm isotropic), 7 T (2 and 1 mm isotropic), and with 100 time points in each case. The 3 T data were acquired with an axial interleaved slice order, number of slices = 56, field of view = 192 mm, TE/TR = 30/3000 ms, flip angle = 90°, phase-encoding R/L, iPat acceleration factor = 2 (GRAPPA reconstruction), and bandwidth = 2111 Hz/Px. The 7 T data (2 mm) were acquired with coronal ascending slice order, number of slices = 27, field of view = 192 mm, TE/TR = 24/2500 ms, flip angle = 90°, phase-encoding S/I, iPat acceleration factor = 2 (GRAPPA), and bandwidth = 1132 Hz/Px. The 7 T 1 mm data were acquired with an increased number of slices (*n* = 54) to retain coverage of the brainstem, iPat acceleration factor = 3 (GRAPPA), and TR = 5000 ms, all other parameters remained the same. All resting fMRI data were analyzed with and without physiological noise modeling (PNM) within the framework of the general linear model in FEAT (part of FSL software, Jenkinson et al., 2012).

Physiological recordings taken with a pulse-oximeter and respiratory bellows, were logged along with scanner volume triggers at 50 Hz sampling using a BIOPAC MP150 (Goleta, CA, USA), and stored on a computer running Acqknowledge 4.2. Cardiac, respiratory, interaction, HR, and RVT regressors were computed for each slice independently using PNM software (part of FSL), giving a total of 34 regressors. Note that no slice timing correction was applied to the data, as the PNM takes into account that each slice is acquired at a specific time, and so slice timing correction, together with the additional interpolation that it performs, is not needed. These PNM regressors were input to the GLM along with a single dummy regressor (a requirement for FEAT), and the model fitted. No smoothing, brain extraction, or temporal filtering was applied, however data were pre-whitened (using FILM, part of FSL) as this was expected to have an effect on physiological sources producing temporal autocorrelation within fMRI time series data (Woolrich et al., 2001). Finally the tSNR was calculated for the raw (motion-corrected only) resting fMRI data and for the residuals following GLM estimation (i.e., with physiological components removed). One important consideration is the loss of DOF incurred when modeling with the PNM, which would be expected to change temporal smoothness and reduce variance even if using a set of randomly generated regressors. To address this the temporal standard deviation (tSTD) was calculated by normalizing with the true DOF, which is equal to the number of time points minus the number of regressors minus one (i.e., *N*−1−*N*_reg_; where *N*_reg_ is equal to 35 in this case – 34 PNM regressors plus 1 dummy regressor).

**Figure 3:**
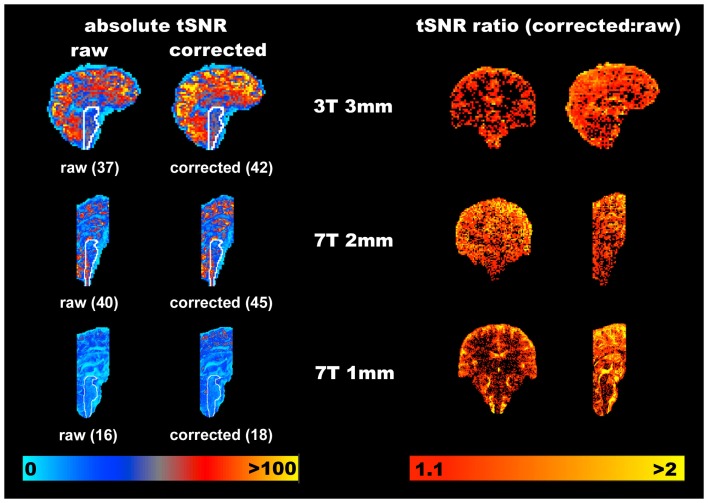
**Representative improvements in temporal signal to noise (tSNR) obtained through modeling physiological noise (single subject data)**. On the left the absolute tSNR maps for each acquisition are provided for both pre- (“raw”) and post-correction (“corrected”), and the corresponding average tSNR within a brainstem mask is given in brackets. Voxels overlapping with the CSF filled spaces around the brainstem benefit most from physiological noise correction. Maps on the right demonstrate the ratio of the corrected to raw tSNR, and are thresholded at 1.1 to indicate where one might reasonably expect to see improvement of greater than 10% in the measured tSNR. Clearly the cortex appears to benefit most from this correction with increases in tSNR of 100% frequently observed at all resolutions and field strengths. The improvement in the brainstem is more modest, but nonetheless improved on average by at least 12.5% in this area (for all acquisitions).

To visualize where in the brain tSNR (temporal mean divided by temporal standard deviation) is increased using the PNM, the absolute tSNR computed for each acquisition before (“raw”) and after correction (“corrected”) is shown in Figure 3, along with the average value within a hand drawn brainstem mask (white outline shows the position of the brainstem). Improvement in tSNR was found for all three acquisitions, but is more easily visualized on the 1 mm isotropic 7 T data, where variance was particularly reduced around the surface of the pons and near the fourth ventricle.

To provide an indication of the spatial localization and magnitude of improvement in tSNR, the ratio of the corrected to the raw tSNR was computed, and is shown using the voxel-wise maps on the right hand side of Figure 3. Whilst there are clearly a significant number of voxels which do not benefit from correction with the PNM, the *average* improvement in tSNR within the brainstem was ∼13% across all three acquisitions.

**Figure 4:**
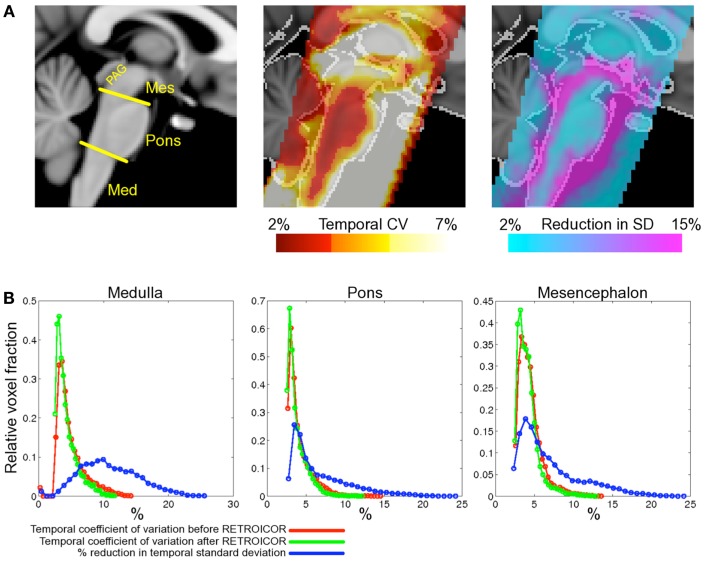
**Spatial localization of brainstem signal variation measured in 12 subjects (adapted from Harvey et al., 2008)**. Top row **(A)** left depicts a midline sagittal slice through the MNI standard brain with the approximate subdivisions of the brainstem indicated (Med, medulla; Mes, mesencephalon; PAG, periaqueductal gray matter). Middle: the mean CV over the group of subjects studied is superimposed to show regions of high signal variability. Highest signal variation was observed in the mesencephalon and near the surface of the pons and medulla. The improvement following application of the modified RETROICOR (3C4R1X) model is demonstrated in the right-most image, which shows the percentage reduction in temporal standard deviation when compared to baseline (no correction). Significant reduction in signal variance was found in and around the PAG and the edges of the pons and medulla. The lower portion of the figure **(B)** shows histograms of the temporal coefficient of variation for both the uncorrected (red) and corrected (green) resting data in the three brainstem regions examined. Voxel counts were normalized to the total number of voxels in each region. Also shown for each region is the histogram of percentage reduction in temporal standard deviation (blue), and demonstrates that the largest benefit of physiological noise modeling occurred in the medulla, where the proportion of voxels with a reduction in SD greater than 10% is largest, although clearly modeling was beneficial in pons and mesencephalon also. (Reproduced from Harvey et al., 2008).

The improvement seen in 3 and 7 T may be compared to that previously reported in the brainstem at 3 T (Harvey et al., 2008), see Figure 4. Harvey et al. studied 12 healthy subjects with a brainstem optimized coronal oblique EPI acquisition (voxel size 2.5 mm × 2.5 mm × 3 mm), TE/TR = 30/1000 ms, flip angle = 70°, to acquire 1130 volumes with simultaneous physiological monitoring (cardiac and respiratory). A series of hierarchical *F*-tests were performed to indicate which physiological regressors (cardiac, respiratory, and interaction) explained significant amounts of noise in their resting data. The final model included three cardiac, four respiratory, and one interaction term (Brooks et al., 2008). The improvement obtained with this model was demonstrated by comparing the temporal coefficient of variation (temporal standard deviation divided by temporal mean, multiplied by 100) between the uncorrected and corrected data. Across the group, temporal CV was reduced most in the medulla, but also near the surface of the pons, near the floor of the fourth ventricle and in mid-brain (particularly around the periaqueductal gray matter, PAG).

## Discussion

There are many available options for dealing with physiological noise, as detailed in the previous section. Deciding which options are best depends very much on the particular experiment being undertaken, the subject cohort, the available equipment, the available expertise, the available software, etc. So one recommendation will not suit all situations. Therefore, in this section we will contrast the various advantages and disadvantages of the different options and suggest a method of piloting and comparing various options in practice.

### Experimental requirements

There are many possible impacts of physiological correction methods on an experiment. One is the extra time in the scanner session required for setup or acquisition. This includes time taken to attach a pulse-oximeter and respiratory bellows (or equivalent devices) to the subject, and verify that signals are recorded correctly. In particular, subjects’ hands should be kept warm to maintain circulation in the fingers, and any nail varnish removed to increase light transmittance; the bellows should be placed carefully near to the diaphragm on the ribcage to maximize changes in recorded lung volume. However, all this can normally be achieved in only a few minutes and has minimal impact on scanning sessions unless timing is very tight. Another impact on scanning time is the addition of new sequences to the session, such as would be required for a resting-state calibration or increased time for certain acquisitions, such as cardiac-gated sequences or certain ME choices. This tradeoff of scan time versus benefit in data quality and scope is a standard problem faced when planning MRI scanning sessions, and will be familiar to experimenters. Evaluating specific benefits of scanning time versus data quality is something that can usually be done with pilot data, as discussed below.

Another requirement for some physiological noise correction methods is extra equipment, such as physiological measuring devices (e.g., pulse-oximeter, respiratory bellows, expired gas analyzer) and digitizing equipment (e.g., BIOPAC, National Instruments, ADInstruments, Cambridge Electronic Devices). When such equipment is not already available, the experimenter must decide whether the additional cost of buying this equipment warrants the benefits to the fMRI analysis. However, given the typical cost of scanning subjects, these pieces of equipment are normally a fairly minor purchase and the monetary cost is rarely a major consideration. Indeed, many MR research centers will typically have patient monitoring apparatus (e.g., *In vivo* Precess, MEDRAD Veris), which can normally output some, if not all, of the required signals (e.g., cardiac triggers) for physiological monitoring. Equally, respiratory monitoring can be achieved with non-magnetic rubber bellows (e.g., from Lafayette Instrument) and commercially available pressure sensors, and data logged with an inexpensive analog to digital converter (e.g., from National Instruments). One essential requirement is that the timing of scan acquisition (i.e., slice or volume triggers) is recorded along with the physiological waveforms using a single computer with a common sampling rate.

Some of the correction methods discussed above also require access to different sequences, such as cardiac-gated sequences, multi-band fMRI, or ME fMRI. These may be more difficult to obtain, being dependent on the type of MR system, requiring input from MR radiographers/operators/physicists and typically a research agreement with the scanner manufacturer. Similarly, other methods require access to raw k-space data and reconstruction algorithms (e.g., RETROKCOR), which can also be difficult to acquire. Therefore, for certain cases some of these correction methods may not be feasible, but given the wide array of options that require nothing more than standard sequences, this is not a major problem.

### Analysis choices and implications

Given the availability of physiological data and analysis routines capable of generating suitable regressors, which should you choose? In our experience the performance of techniques based on “binning” time series data according to slice and physiological waveform timing (e.g., Deckers et al., 2006), depend critically on the choice of total number of bins (Kong et al., 2012), and do not address issues relating to depth of breathing, which can be critical. ICA approaches offer the possibility to automatically identify sources on physiological noise, however, their usefulness must be judged against the possibility of true signal being identified as a noise source and removed from the data. One approach to mitigate this outcome is to acquire resting and task fMRI data, and use the resting data to define physiological noise components, for subsequent removal from the task data (e.g., Xie et al., 2012).

Concerning the model-based techniques, such as RETROICOR (Hu et al., 1995; Josephs et al., 1997; Glover et al., 2000) and PNM (Brooks et al., 2008; Harvey et al., 2008) these will always be subject to the limitations of the basis functions used to describe the physiological processes they are attempting to model. Further developments that attempt to capture variance induced by changes in HR (e.g., Shmueli et al., 2007; Chang et al., 2009) or respiratory rate and depth (e.g., RVT, Birn et al., 2006) will be necessary to account for effects not accounted for in RETROICOR or PNM. These additional regressors are straightforward to compute, and mostly likely account for low frequency signal fluctuations in time series fMRI data. There are however, additional considerations one must take into account when using these slice dependent model-based approaches. For example, slice timing correction when applied to time series fMRI data could potentially break the association between the physiologically induced signal changes and signal changes predicted by the model. Equally, with increased use of multiplexed parallel imaging, e.g., multi-band (Feinberg et al., 2010) or simultaneous multi-slice imaging (Setsompop et al., 2012), researchers will need to be careful in assigning cardiac and respiratory phases to subsets of their slices acquired at the same time. For 3D or segmented acquisitions, it may be more appropriate to perform corrections on raw k-space data (Tijssen et al., in press). Finally, one must be aware of the penalty in terms of loss of DOF incurred when using these model-based techniques. However, the loss of 40 DOF within the context of a time series of 100 or more time points will constitute a relatively small change in statistics, with potentially greater ability to detect signal against the background noise in the experiment.

### Optimizing your experiment

Given the many ways in which different experiments and experimental setups can vary it is often necessary to decide on the best strategy for acquisition and analysis for each experiment separately. Collecting specific pilot data is usually the best way to make this decision; however evaluating small pilot experiments is not straightforward. Here we offer some advice on how to collect and evaluate pilot data in order to evaluate different acquisition and analysis choices. It cannot be guaranteed that this will lead to the best possible experiment, but following a procedure like this is very likely to improve the experimental results.

Certain types of experiment can be more demanding in terms of finding effects of interest, such as pain experiments where subjects often move more and there may be correlations between the applied stimulus and physiological changes in respiratory and cardiac cycles (e.g., Tousignant-Laflamme et al., 2005; Kong et al., 2009). Furthermore, for brainstem investigations that involve neural mechanisms that play a role in respiratory control (Smith et al., 2013b) or autonomic regulation (Macefield and Henderson, 2010), it will be very difficult to disentangle BOLD-related activations and physiological noise and therefore extra care and attention to the analysis is called for (Pattinson et al., 2009a,b).

Once the limitations and difficulties have been thought about and the various pros and cons of the correction methods have been weighed up, it is normally necessary to evaluate a limited number of alternative options. For example, using a cardiac-gated sequence or not, or applying a measurement-driven method like RETROICOR versus ICA denoising. If multiple options are being considered, it is usually beneficial to rank comparisons in order of importance and then conduct a number of separate pairwise comparisons, rather than trying to evaluate the full factorial set of options, since the latter usually ends up taking an enormous amount of time.

When comparing different acquisition methods, it is sufficient to compare the tSNR directly, provided that the basic contrast mechanism is the same; for example, both using BOLD contrast. However, the comparison should not be as simple as “larger is better” when there are changes in pre-processing (e.g., different amounts of spatial smoothing) or statistical estimation. This is because it would lead to the conclusion that increasing the filtering, or number of regressors, or number of components in denoising, is always beneficial, even though this is not the case. Instead, the relative benefit of including extra regressors, or changing the pre-processing options, should be evaluated using techniques such as *F*-tests or information-theory measures like the Bayesian Information Criterion (BIC). These techniques are based on probability theory and take into account the fact that random noise is always soaked up by extra filtering, denoising, or regression, but that removing too much of the thermal noise makes it more difficult to estimate the statistics reliably, thus decreasing the statistical DOF, which would lead to weaker, not stronger, statistics.

Calculation of the *F*-test is straightforward for methods that are embedded within the same statistical estimation framework. For example, testing whether a set of additional regressors in RETROICOR is beneficial can be done using an *F*-test over the extra regressors (e.g., as done in Brooks et al., 2008; Harvey et al., 2008). This will give a statistical result at each voxel that tests whether these extra regressors fit an amount of variance that is beyond what would be expected from thermal noise alone. The overall benefit can then be judged subjectively, based on whether a sufficient number of voxels in the area of interest show a significant statistical result or not.

When comparing differing pre-processing steps it is more difficult to use *F*-tests, and this is where the BIC becomes more useful. For example, the BIC for k regressors and N time points is BIC(*k*, *N*) = *N* × log(RSS/*N*) + *k* × log(*N*), where RSS is the residual sum of squares. Therefore, comparing models with k_1_ and k_2_ regressors, which can be from very different sets, yields the condition that log[RSS(*k*_1_)/RSS(*k*_2_)] < (*k*_2_ − *k*_1_) × log(*N*)/*N* when the model with k_1_ regressors is considered superior. This test again needs to be performed separately on each voxel and then the overall result can be judged subjectively, based on the pattern of results. In cases where the correction method is not a straightforward regression, an approximate number of regressors can be used, based on the number of denoised components or the rank of the matrix used to perform filtering.

## Conclusion

In this Methods Article we have presented several different techniques for estimating and removing physiological noise artifacts from time series fMRI data. It is clear that the brainstem suffers from both intrinsically low signal to noise, and increased contributions from non-neuronal physiological sources, which serve to reduce the temporal SNR. The recorded temporal SNR in any given brain region is an important consideration as it will, to a large extent, dictate the length of an fMRI experiment required to measure signals of interest (Murphy et al., 2007). Physiological noise models, and denoising techniques in general, aim to reduce signal variation in the fMRI time series and increase sensitivity to detect activation. From considerations of the effects of field strength and voxel size on recorded tSNR, it is clear that to benefit from the increased BOLD signal afforded by higher field strength MRI systems (3 T and above), one should make a considered choice about the resolution of acquired images. Minimizing voxel size will reduce the influence of physiological noise, but the “intrinsic” signal to noise will be low. This places a large emphasis on choosing the correct voxel dimensions for your experiment, which will inevitably be a compromise between visualizing the small brainstem structures, whilst retaining sensitivity to neuronally induced BOLD changes.

Table 1 provides the URLs for several different packages used for removing or correcting for the effects of physiological noise.

**Table 1:** **Summary of available physiological noise correction/removal tools**.

Tool	Requirements	URL
RETROICOR (RETROspective Image CORrection)	Part of AFNI	http://afni.nimh.nih.gov/pub/dist/doc/program_help/3dretroicor.html
RETROICOR	Freesurfer/Matlab	https://github.com/neurodebian/freesurfer/blob/master/fsfast/toolbox/fast_retroicor.m
DRIFTER (Dynamic RetrospectIve FilTERing)	SPM (Toolbox)	http://becs.aalto.fi/en/research/bayes/drifter/
PNM (physiological noise model)	Part of FSL	http://fsl.fmrib.ox.ac.uk/fsl/fslwiki/PNM
**STANDALONE PACKAGES**		
PART (Physiological Artifact Removal Tool)	Windows executable	http://www.cabiatl.com/CABI/resources/part/
PhLEM (Physiological Log Extraction for Modeling)	Requires Matlab	https://github.com/timothyv/Physiological-Log-Extraction-for-Modeling--PhLEM--Toolbox
RETROICOR tool	Requires Matlab	http://cbi.nyu.edu/software/
PhysioNoise	Requires Python	http://www.plosone.Org/article/info:doi/10.1371/journal.pone.0001751#pone.0001751.s001
PhyslO Toolbox	Requires Matlab	http://www.translationalneuromodeling.org/tnu-checkphysretroicor-toolbox/
**ICA-BASED**		
FIX (FMRIB’s ICA-based X-noisifier)	Part of FSL	http://fsl.fmrib.ox.ac.uk/fsl/fslwiki/FIX
PESTICA (Physiologic EStimation by Temporal ICA)	Requires Matlab	http://www.nitrc.org/projects/pestica
ICA Artifact Remover NIAK (NeuroImaging Analysis Kit)	Requires Matlab	http://www.cs.tut.fi/~jupeto/software.html
	Requires Matlab/octave	https://code.google.com/p/niak/

Please note that this list should not be considered to be definitive. It is provided to give some indication of the available software, and does not constitute a recommendation or endorsement.

## Conflict of Interest Statement

The authors declare that the research was conducted in the absence of any commercial or financial relationships that could be construed as a potential conflict of interest.
